# A New Grading System for Migrated Lumbar Disc Herniation on Sagittal Magnetic Resonance Imaging: An Agreement Study

**DOI:** 10.3390/jcm11071750

**Published:** 2022-03-22

**Authors:** Yong Ahn, Ji-Eun Kim, Byung-Rhae Yoo, Yu-Mi Jeong

**Affiliations:** 1 Department of Neurosurgery, Gachon University Gil Medical Center, Incheon 21565, Korea; byungryoo@gilhospital.com; 2 College of Medicine, Gachon University, Incheon 21565, Korea; 201739567@medicine.gachon.ac.kr; 3 Department of Radiology, Gachon University Gil Medical Center, Incheon 21565, Korea; youme34@gilhospital.com

**Keywords:** agreement, grade, lumbar, migrated disc herniation, magnetic resonance imaging

## Abstract

Understanding the degree of disc migration is essential in order to diagnose, treat, and assess the prognosis of migrated lumbar disc herniation (LDH). Based on anatomical configuration, we developed a simple six-level grading system for migrated lumbar disc herniation. We aimed to evaluate whether the new grading system was reliable and could replace the previous grading system. We selected 101 cases from our database. Two independent raters evaluated the magnetic resonance images using each grading system. Interobserver, intraobserver, and inter-grading system agreements were assessed using kappa statistics. The most common migration pattern was low-grade inferior migration. Interobserver agreements between the two readers showed substantial agreement in the first and second assessments (k = 0.753 and 0.756, respectively). The intraobserver agreement of reader 1 revealed substantial agreement (k = 0.733), while that of reader 2 revealed almost perfect agreement (k = 0.829). The strengths of the agreements of the new grading system were higher than those of the Lee-Kim grading system. The two grading systems agreed almost perfectly for most measurements. The new grading system was reliable and feasible to determine migrated LDH grade. It allowed for a more intuitive, objective measurement and helped select surgical options.

## 1. Introduction

The current standard surgical option for lumbar disc herniation (LDH) is open microdiscectomy or minimally invasive endoscopic lumbar discectomy [1,2,3,4,5]. The herniated disc fragment can be easily removed when the piece is near the maternal disc. However, a remotely located fragment may be challenging to remove in the case of a migrated LDH in the sagittal plane. Migrated LDH refers to the displacement of the herniated disc material from the annular opening through which the disc is disrupted [6]. Properly classifying the degree of disc migration may be essential to determine an appropriate surgical method to achieve the best clinical outcome for a patient [7,8,9,10,11]. Regarding a non-migrated extruded LDH, an endoscopic discectomy may be effective because of its minimal invasiveness and quick recovery time [12,13,14]. In contrast, in the case of highly migrated LDH, an interlaminar microdiscectomy may be better than a transforaminal endoscopic approach [9,10].

Several grading systems based on magnetic resonance imaging (MRI) have been presented to determine the grade of disc migration—low, high, and very high grades. Four-level [10,15,16] or six-level [17,18,19] grading systems of migrated LDH have been reported. There has been a clear consensus among various grading systems of the baseline for very high-grade migration as the inferior margin of the pedicle [17,18,19]. However, the baseline for the more clinically important high-grade migration has been debatable. Lee et al. presented a four-zone grading system, setting the baseline for high grade as 3 mm below the lower pedicle margin [10]. Lee et al.’s grading system was then modified by Kim et al. into a six-level grading system, in which the baseline for high grade was the height of the posterior marginal disc space [17]. Both grading systems are limited because they are complicated to measure and do not account for each patient’s variable disc heights and uneven disc spaces.

We suggested a six-level grading system and evaluated its reliability based on the Lee–Kim classification [10,17,19]. The grading system was found to have good to excellent intraobserver and interobserver agreement. However, there may be some problems with the clinical application of the grading system: (1) it is not based on the anatomical structures; (2) the value 3 mm was arbitrarily determined based on the authors’ experience; (3) there may be some deviation (error) in measuring the 3 mm on an MRI.

Therefore, a modified grading system that is based on anatomical landmarks and is easily measurable is required. We changed the grading system for the migrated LDH into another six-level grading system, to determine whether it may provide a simpler and more direct measurement, tailored to the anatomical differences of each patient. Therefore, the objectives of this study were to evaluate the reliability of the modified grading system for migrated LDH and discuss its clinical relevance.

## 2. Materials and Methods

### 2.1. New Magnetic Resonance Imaging Grading System for Migrated Lumbar Disc Herniation

**Figure 1:**
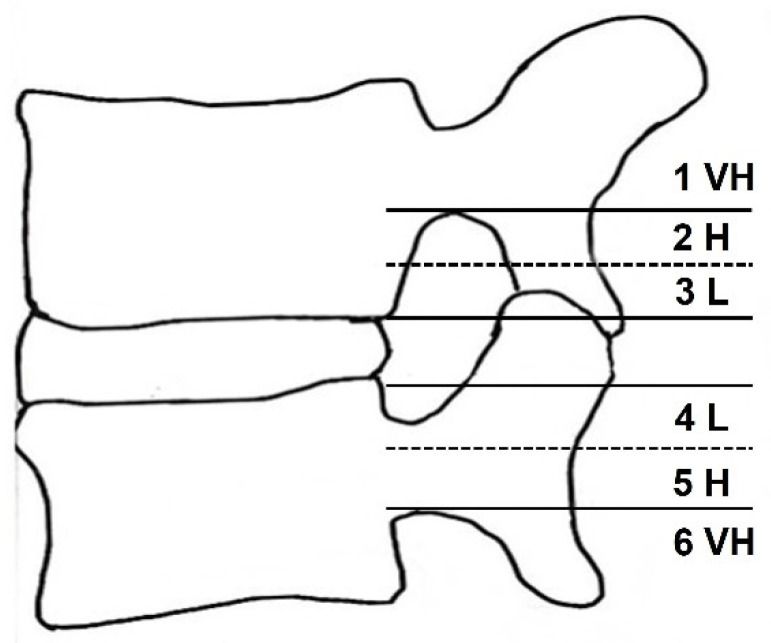
Schematic illustration of the six migrated lumbar disc grades of the new grading system in the sagittal plane. Note the degree of disc migrations; very high (VH), high (H), and low (L).

Migrated LDH was classified into six grades, based on the distance from the maternal disc and the direction on T2-weighted sagittal MRI [10,16,17,18]. The very high grade was defined as disc migration beyond the inferior margin of the upper pedicle (superior migration) or the lower pedicle (inferior migration), as in the previous grading system (Lee–Kim grading system) [10,17,18]. The high grade was defined as disc migration beyond the midpoint between the inferior margin of the upper pedicle and superior disc margin (superior migration) or the midpoint between the inferior margin of the lower pedicle and inferior disc margin (inferior migration). The low grade was defined as short migration beyond the superior disc margin (superior migration) or the inferior disc margin (inferior migration). Because each patient’s disc and pedicle size are different, this new grading system uses the midpoint as a new reference point rather than the 3 mm used in the Lee–Kim system (Table 1 and Figure 1).

### 2.2. Study Population

A total of 101 consecutive cases of migrated LDH were enrolled in this study, from a surgical database. The eligibility criterion was a single-level, migrated LDH with different distances of disc migration on the sagittal plane, regardless of the continuity with the maternal disc. Cases of non-migrated LDH, concurrent central stenosis or foraminal stenosis, intradural LDH, and other pathological conditions, such as infection, fracture, tumor, or painless weakness, were excluded from the study. The institutional review board approved the study, and informed consent was not required to analyze the magnetic resonance (MR) images.

### 2.3. Image Measurement and Analysis

**Figure 2:**
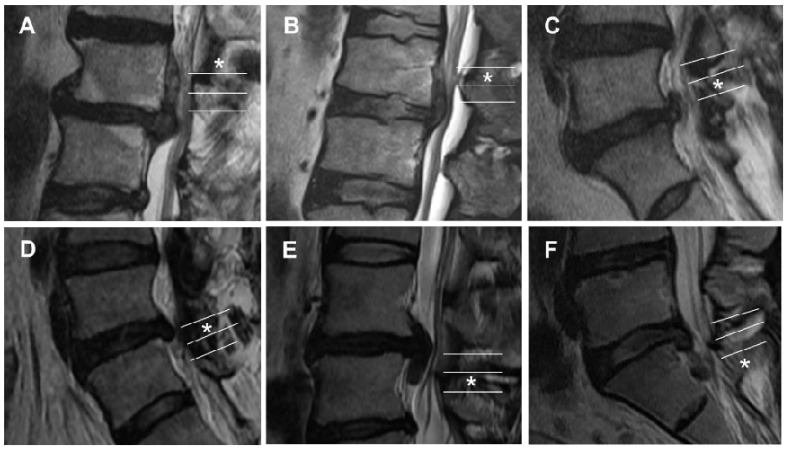
Grade of migrated lumbar disc herniation on a sagittal MRI image (asterisk). (**A**) Grade 1, superior very high grade. (**B**) Grade 2, superior high grade. (**C**) Grade 3, superior low grade. (**D**) Grade 4, inferior low grade. (**E**) Grade 5, inferior high grade. (**F**) Grade 6, inferior very high grade.

Image measurements were performed using T2-weighted sagittal MRI (Magnetic resonance imaging) images (repetition time/time to echo, 4010/105; slice thickness, 4 mm; slice gap, 0.4 mm; matrix, 512 × 307; field of view, 30 cm; flip angle, 90°; the number of signal averages, 2). Values were automatically calculated using an electronic cursor via commercially available software (PiView Star; INFINITT, Seoul, Korea). Two blinded observers independently measured the degree of disc migration in the 101 cases using the Lee–Kim and new grading systems (Figure 2). Each reader evaluated the MRI images twice, and a second evaluation was performed 3 months after the first measurement.

### 2.4. Statistical Analysis

For the MRI grading evaluations, statistical analyses were performed to obtain the kappa value (k) using SPSS Statistics for Windows, version 18.0 (SPSS Inc., Chicago, IL, USA). The level of significance was set at *p* < 0.05. In this study, k was used to interpret the strength of agreement between the two readers (interobserver agreement) and between the two evaluations of each reader (intraobserver agreement): poor (k < 0), slight (0 ≤ k ≤ 0.2), fair (0.2 < k ≤ 0.4), moderate (0.4 < k ≤ 0.6), substantial (0.6 < k ≤ 0.8), and almost perfect (0.8 < k ≤ 1) [20].

## 3. Results

### 3.1. Demographics

The 101 patients included 43 women and 58 men, with a mean age of 45.93 ± 14.59 (range, 17–79) years. The levels of migrated LDH were L1–L2 in 1 (0.99%) patient, L2–L3 in 3 (2.97%) patients, L3–L4 in 17 (16.83%) patients, L4–L5 in 51 (50.50%) patients, and L5–S1 in 29 (28.71%) patients. The migrated LDH in the sagittal plane was superior in 47 (46.53%) patients and inferior in 54 (53.47%) patients. The patient demographics are summarized in Table 2.

### 3.2. Distribution

The distribution of migrated LDH, based on the migration grade by the two observers, is presented in Table 3. The most common grade was low-grade inferior migration (grade 4, 25.99%), followed by high-grade inferior migration (grade 5, 20.30%). The rarest grade was a very high-grade inferior migration type (grade 6, 7.43%). No statistical difference was observed among the grades (*p* = 0.9527).

**Figure 3:**
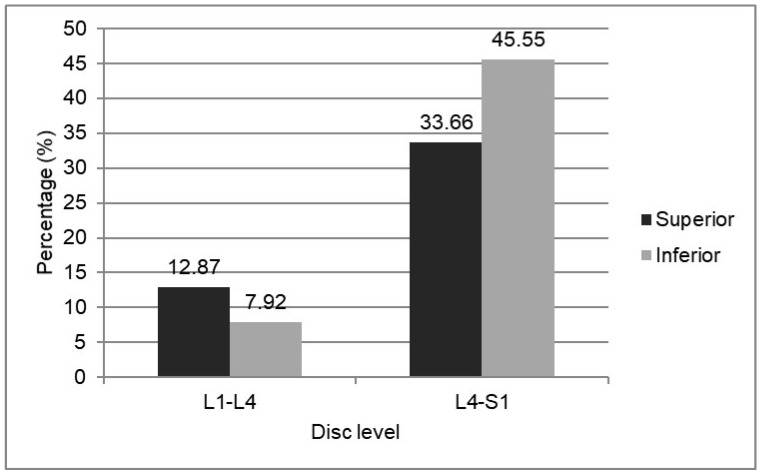
The direction of disc migration in the sagittal plane stratified by the level of disc herniation. Note the tendency for more superior migration in the upper lumbar disc level and more inferior migration in the lower (*p* < 0.05).

The direction of disc migration was found to differ according to the disc level or the patient’s age. Although it did not reach statistical significance, the superior disc migration tended to be higher in the upper lumbar level (61.9% in the L1–2, L2–3, or L3–4 levels), while the inferior disc migration tended to be higher in the lower lumbar level (57.5% in the L4–5 or L5–S1 level; *p* = 0.1426, Figure 3).

**Figure 4:**
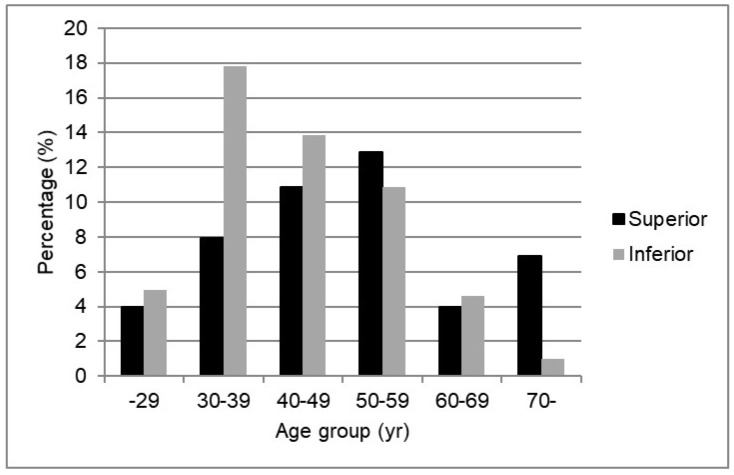
The direction of disc migration in the sagittal plane stratified by age. Note the tendencies of more superior migration in the patients older than 50 years of age and more inferior migration in the patients younger than 50 years of age (*p* = 0.0671).

There was a tendency for more superior disc migration in older patients aged ≥ 50 years (58.5%), whereas there was a tendency for more inferior disc migration in younger patients aged < 50 years (61.7%; *p* = 0.0671, Figure 4).

### 3.3. Interobserver and Intraobserver Agreements

The interobserver agreements of the two readers showed substantial agreement in both the first and second assessments (k = 0.753 and 0.756, respectively). The intraobserver agreement of reader 1 revealed substantial agreement (k = 0.733), while that of reader 2 revealed almost perfect agreement (k = 0.829). The interobserver and intraobserver agreements, using the new grading system, tended to be higher than those using the Lee–Kim grading system (Table 4 and Table 5).

### 3.4. Inter-Grading System Agreement

The agreement between the two grading systems was almost perfect for most measurements. The k values of the first and second measurements by reader 1 were 0.878 and 0.779, respectively, indicating an almost perfect and substantial inter-grading system agreement. The k values of the first and second measurements by reader 2 were 0.828 and 0.841, respectively, indicating an almost perfect inter-grading system agreement (Table 6). All kappa values were statistically significant with a *p* < 0.05.

## 4. Discussion

### 4.1. Comparison of the Grading Systems

Determining the correct grade of disc migration is crucial in selecting the best surgical treatment and improving treatment outcomes. Currently, there is no clear consensus on which grading system is the most effective in defining the degree of disc migration. To our knowledge, there is no study comparing the grading systems of migrated LDH [10,15,16,17,18,19]. The present study presents a new grading system that proves to be as reliable as the Lee–Kim grading system [19]. The Lee–Kim grading system is a six-level grading system combining the Lee et al. [10] and Kim et al. systems [17,18]. The reliability of the Lee–Kim grading system was proven by an agreement study [19]. It may be helpful for classifying the degree of disc migration in various clinical situations. However, this system has some limitations. First, it may be difficult to accurately measure a 3 mm distance from the pedicle margin, and it does not consider the variable pedicle size among different patients. Therefore, the measurement may be time-consuming and may result in numerous errors.

The new grading system uses the midpoint between the pedicle margin and disc margin, thereby overcoming the limitations of the Lee–Kim grading system and making intuitive and objective measurements possible. The new grading system has several benefits compared with the previous method. First, the new grading system is based on objective anatomical landmarks. Therefore, it can guarantee consistent and reliable measurements. Second, it is easier and quicker to assess the degree of disc migration. Finally, this system can be applied to other imaging studies, such as computed tomography scans or myelograms.

Our data verified the theoretical benefits of the new grading system. The level of reliability of the new system was not inferior to that of the Lee–Kim grading system [19]. The k values of the new process were higher than those of the previous one in both the interobserver and intraobserver agreements. We can interpret this result as indicating that the new grading system may be easier to record measurements and consistent for application in the clinical evaluation of migrated LDH.

### 4.2. Distribution

In the current study, the most common grade of LDH was grade 4, followed by grades 5 and 1 (Table 3). This does not reflect the actual incidence of migrated LDH, but the incidence of surgically indicated cases. Regarding the inferiorly migrated LDH, the incidence of operated cases showed a sequential distribution from lower to higher grades. However, regarding the superiorly migrated LDH, the most common type of surgical indication was very high-grade migration. This means that superiorly migrated LDH is rarely indicated for surgery, and only patients with severely migrated may be candidates for surgery.

According to previous studies, the direction of disc migration may be related to the level of LDH or patient age. The rate of superior migration has been reported to be high in the upper lumbar level and in older patients [19,21]. This may be related to the aging process in the anterior epidural space at the upper lumbar disc and in elderly patients [21,22]. However, the data did not show statistically significant results, and we could not conclude or generalize that the direction of disc migration is related to the patients’ age. Therefore, we postulate that the clinical manifestation may be affected by accompanying disease or degenerative changes over time. Further studies on the incidence and characteristics of migrated LDH are warranted.

### 4.3. Clinical Usefulness of the Grading System of Migrated Lumbar Disc Herniation

The new migration grading system is clinically important. First, it may be helpful to categorize the clinical features of migrated LDH, including neurologic deficits, manifested symptoms, and natural course based on the degree of disc migration. Second, it may offer an excellent criterion to determine an appropriate surgical option for migrated LDH. Minimally invasive or endoscopic procedures can be compared with standard microdiscectomy techniques. The surgeon can set an adequate surgical plan using various surgical tools. The treatment prognosis of different surgical options can also be expected according to the degree of disc migration.

### 4.4. Limitations

There are a couple of limitations to our study. First, the current data are limited to migrated LDH that has been surgically treated and does not include cases that have not been surgically treated or non-migrated LDH. Therefore, it does not reflect the actual incidence of migrated disc herniation in the entire population. Second, the new grading system is simply an anatomical classification and does not consider the level of surgical difficulty or prognosis of the surgery. Further studies are needed to determine the prediction rules and selection of the surgical approach according to the disc migration grade and which treatment options are best suited to the given conditions.

## 5. Conclusions

The new grading system for migrated LDH is reliable and practical. Thus, it can be used to compare the surgical outcome according to the grade of disc migration and determine suitable treatment options for migrated LDH.

## Figures and Tables

**Table 1:** A new grading system for migrated lumbar disc herniation.

Grade	Direction and Degree	Range of Migration Distance
1	Superior very high	Beyond the inferior margin of the upper pedicle
2	Superior high	From the inferior margin of the upper pedicle to the midpoint between the inferior margin of the upper pedicle and superior disc margin
3	Superior low	From the midpoint between the inferior margin of the upper pedicle and superior disc margin to the superior disc margin
4	Inferior low	From the inferior disc margin to the midpoint between the inferior margin of the lower pedicle and inferior disc margin
5	Inferior high	From the midpoint between the inferior margin of the lower pedicle and inferior disc margin to the inferior margin of the lower pedicle
6	Inferior very high	Beyond the inferior margin of the lower pedicle

**Table 2:** Demographic data of patients with migrated lumbar disc herniation.

Characteristic	No.
Patients	101
Sex	
Female	43 (42.57%)
Male	58 (57.43%)
Age (years)	45.91 ± 14.64
Age group (years)	
≤29	9 (8.91%)
30–39	26 (25.74%)
40–49	25 (24.75%)
50–59	24 (23.76%)
60–69	9 (8.91%)
≥70	8 (7.92%)
Level of migrated LDH	
L1–L2	1 (0.99%)
L2–L3	3 (2.97%)
L3–L4	17 (16.83%)
L4–L5	51 (50.50%)
L5–S1	29 (28.71%)
Direction of disc migration	
Upward (superior)	47 (46.53%)
Downward (inferior)	54 (53.47%)

LDH, lumbar disc herniation; no., number.

**Table 3:** Distribution of migrated lumbar disc herniation by observers.

	Grade 1	Grade 2	Grade 3	Grade 4	Grade 5	Grade 6	Total
Reader 1 (first)	17	13	17	27	16	11	101
Reader 1 (second)	18	16	13	28	19	7	101
Reader 2 (first)	14	14	18	26	23	6	101
Reader 2 (second)	17	17	13	24	24	6	101
Total	66	60	61	105	82	30	404
%	16.34	14.85	15.10	25.99	20.30	7.43	

**Table 4:** Interobserver agreement.

Observer	Grading System	k (95% CI)	Agreement
Reader 1 vs. 2 (first)	New	0.753 (0.658–0.848)	Substantial
	Lee–Kim	0.714 (0.614–0.814)	Substantial
Reader 1 vs. 2 (second)	New	0.756 (0.660–0.852)	Substantial
	Lee–Kim	0.742 (0.645–0.840)	Substantial

Strength of agreement: poor (k < 0), slight (0 ≤ k ≤ 0.2), fair (0.2 < k ≤ 0.4), moderate (0.4 < k ≤ 0.6), substantial (0.6 < k ≤ 0.8), and almost perfect (0.8 < k ≤ 1). k, kappa value; CI, confidence interval; vs., versus.

**Table 5:** Intraobserver agreement.

Observer	Grading System	k (95% CI)	Agreement
Reader 1	New	0.733 (0.636–0.831)	Substantial
	Lee–Kim	0.729 (0.630–0.828)	Substantial
Reader 2	New	0.829 (0.747–0.912)	Almost perfect
	Lee–Kim	0.668 (0.564–0.773)	Substantial

Strength of agreement: poor (k < 0), slight (0 ≤ k ≤ 0.2), fair (0.2 < k ≤ 0.4), moderate (0.4 < k ≤ 0.6), substantial (0.6 < k ≤ 0.8), and almost perfect (0.8 < k ≤ 1). k, kappa value; CI, confidence interval.

**Table 6:** Inter-grading system agreement.

Observer	Grading System	k (95% CI)	Agreement
Reader 1	New vs. Lee–Kim (first)	0.878 (0.807–0.950)	Almost perfect
	New vs. Lee–Kim (second)	0.779 (0.686–0.872)	Substantial
Reader 2	New vs. Lee–Kim (first)	0.828 (0.744–0.911)	Almost perfect
	New vs. Lee–Kim (second)	0.841(0.760–0.921)	Almost perfect

Strength of agreement: poor (k < 0), slight (0 ≤ k ≤ 0.2), fair (0.2 < k ≤ 0.4), moderate (0.4 < k ≤ 0.6), substantial (0.6 < k ≤ 0.8), and almost perfect (0.8 < k ≤ 1). k, kappa value; CI, confidence interval.

## Data Availability

The data used to support the findings of this study are available from the corresponding author upon request.
